# Carbon ion radiotherapy for desmoid tumor of the abdominal wall: a case report

**DOI:** 10.1186/s12957-016-1000-8

**Published:** 2016-09-13

**Authors:** Takuya Nagata, Yusuke Demizu, Tomoyuki Okumura, Shinichi Sekine, Naoki Hashimoto, Nobukazu Fuwa, Tomoaki Okimoto, Yutaka Shimada

**Affiliations:** 1 Department of Surgery and Science, Graduate School of Medicine and Pharmaceutical Sciences for Research, University of To1yama, 2630 Sugitani, Toyama, 930-0194 Japan; 2 Department of Radiology, Hyogo Ion Beam Medical Center, 1-2-1, Koto, Singu, Tatsuno, Hyogo 679-5165 Japan; 3 Department of Radiation Oncology, Kobe Minimally Invasive Cancer Center, 8-5-1Minatojima, Tyuoku, Kobe, Hyogo 650-0046 Japan; 4 Department of Radiation Oncology, Ise Red Cross Hospital, 1-471-2 Funae, Ise, Mie 516-8512 Japan; 5 Department of Nanobio Drug Discovery, Graduate School of Pharmaceutical Sciences, Kyoto University, 46-29 Shimoadachi, Yoshida, Sakyoku, Kyoto, 606-8501 Japan

**Keywords:** Desmoid tumor, Mesenteric, Unresectable, Carbon ion radiotherapy

## Abstract

**Background:**

Desmoid tumors, which are associated with familial adenomatous polyposis (FAP), tend to occur frequently in the abdominal wall and mesentery. Currently, there are no recognized treatments other than surgery, and frequent surgeries result in gastrointestinal obstructions and functional gastrointestinal disorders.

**Case presentation:**

After surgery that was performed on a 39-year-old patient with FAP, we performed a second tumor excision which was the procedure used for frequently occurring mesenteric desmoid tumors. It was determined that the enlarged tumor would be difficult to operate on through an abdominal incision. Subsequently, the carbon ion radiotherapy of 50 Gy was then performed on the patient. Three years later, the tumor still remains reduced in size. In addition, we have not observed any negative effect on the digestive tract.

**Conclusions:**

This is the first instance that the carbon ion radiotherapy has been effective for the unresected desmoid tumor, and it is believed that this will become the one effective option for the treatment of desmoid tumors.

## Background

Desmoid tumors associated with familial adenomatous polyposis (FAP) are classified as benign, but clinically considered to be a disease similar to malignancies because of their tendency to occur as multiple lesions in the abdominal wall or mesentery. Surgical therapy is the first choice of treatment, but repeated procedures for recurrent tumors may cause gastrointestinal stenosis or dysfunction. Although hormone therapy and chemotherapy for desmoid tumors have been described in the literature, no established treatment is available other than surgical resection. We report a case of inoperable growing desmoid tumor in the abdominal wall that shrank in response to heavy ion radiotherapy and has remained stable and free of effects on the adjacent intestinal tract for 3 years. No cases like this have been reported anywhere in the world. We believe that heavy-ion radiotherapy has the potential to become an effective treatment option for patients with desmoid tumors.

## Case presentation

**Fig. 1:**
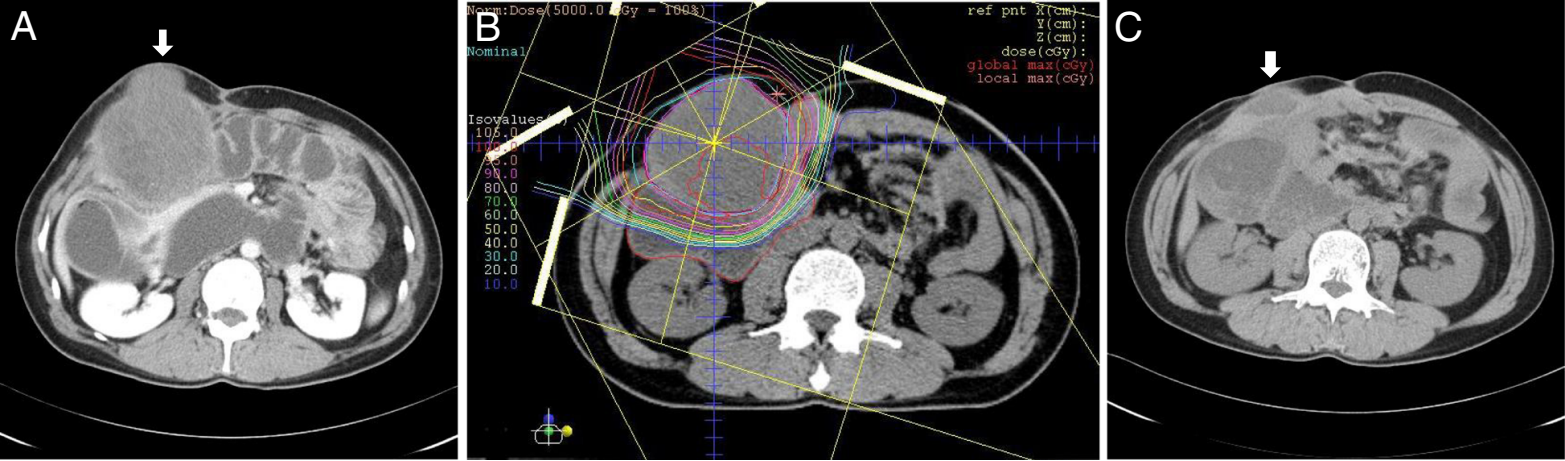
Abdominal computed tomography (CT) scan. CT scan revealed the desmoid tumor in abdominal wall (*solid white arrow* in **a** and **c**). **a** Pretreatment phase, **b** Carbon ion treatment plan and **c** 3 years after ion radiotherapy were shown

The patient was a 49-year-old man diagnosed with familial adenomatous polyposis (FAP) caused by a defect in the adenomatous polyposis coli (APC) gene. In March 1998 (aged 36), he underwent total proctocolectomy and ileostomy for polyps involving the entire colon, and the stoma was closed 9 months later. Pathological findings showed that the polyps were adenomas. In August 2001 (aged 39) and August 2003 (aged 41), multiple mesenteric tumors were removed, which were pathologically diagnosed as desmoid tumors characterized by invasive growth of fibroblasts into the surrounding muscle layer. He subsequently experienced repeated episodes of ileus symptoms attributable to intestinal adhesion as a postoperative complication and to recurrent mesenteric desmoid tumors; thus, duodenojejunostomy was performed in March 2011 (aged 49). Around December 2011, 9 months after the operation, a desmoid tumor of the abdominal wall was found immediately above the peritoneum just below the previous operative wound, which grew to 7.3 cm in the longest diameter in 3 months (Fig. 1a). It was considered surgically unresectable partly because tumor adhesion to the intestinal tract and peritoneum was severe and partly because tumor removal would have resulted in a large defect in the abdominal wall. Among the available treatment options for desmoid tumor of the abdominal wall, chemotherapy, carbon ion radiotherapy, etc. were proposed. After a thorough discussion about informed consent, the patient selected carbon ion radiotherapy, which is a type of radiotherapy that can deliver high-dose radiation to a tumor while minimizing the dose delivered to the organs at risk. From May to July 2012, he received carbon ion beam irradiation at a total dose of 50 Gy (relative biological effectiveness [RBE])^1^ in 25 fractions at the Hyogo Ion Beam Medical Center (HIBMC). Figure 1b shows the carbon ion treatment plan for this patient. The solid abdominal wall tumor continued to grow until 2 months after treatment, reaching 12 cm in diameter, and then gradually shrank to 7.8 cm at 5 months. One year after treatment, the contents of the tumor had liquefied, and the tumor had shrunk to 4.3 cm in diameter. The liquid components consisted primarily of fibrin, with no malignant cells. Currently, 3 years after the operation, the abdominal wall tumor remains stable, with a diameter of 3.3 cm, and is being followed up (Fig. 1c).

### Discussion

Desmoid tumor is a rare with 2.4~4.3 cases occurring per one million individuals each year [1]. It has been correlated with the FAP [2], and the overall incidence of desmoid tumor in patients with FAP is 10–20 %, which equates to relative risk of 852 compared to the general population [3]. Desmoid tumor is a benign fibromatosis that originate from fascia and muscular aponeuroses, with an infiltrating growth pattern. As indicated by its name, aggressive fibromatosis, a desmoid tumor is characterized by a clinically malignant course, with a reported recurrence rate up to 45 % after surgical resection [4].

Treatment of desmoid tumor is a complex condition with many recognized treatments including active observation, hormonal therapy, nonsteroidal anti-inflammatory drugs (NSAIDs), chemotherapy, radiotherapy, and surgical resection [5]. As desmoid tumors sometimes exhibit characteristics of stable or regress, the conservative approach is recommended [6]. In this case, the tumor grew more than 7 cm in just 3 months and the patient complained the tumor pain, so it was necessary to provide the aggressive treatments. Radiotherapy has been adopted as a primary treatment in patients who undergo progression during observation. Mounting literature supports this approach, with several groups reporting long term local control in 70–93 % of patients at doses of 50–60 Gy (1.8–2 Gy/fraction) [7, 8]. It is also reported that the radiotherapy combined with surgical resection results in better progression-free survival time (PFS) than surgery alone [9]. But simultaneously, radiotherapy has a risk to cause damage to the neighboring intestine and organ such as perforation or abscess formation, and potential late radiation effects, including second malignancies [10]. It was reported a partial response of hormone therapy (tamoxifen) for desmoid tumor in male patients, 24~54 months after treatment initiation as well as female patients [11]. And, there is some evidence that the treatments are most effective when NSAIDs and hormonal therapies are given together [12]. Various chemotherapy regimes were reported and most of them were used as combination, included methotrexate with vinblastine or vinorelbine, doxorubicin with dacarbazine, doxorubicin with cyclophosphamide and vincristine, and actinomycin-D-based chemotherapy. The average response rate of them was 50 % (range 17~100 %) [13]. Although hormonal therapies, nonsteroidal anti-inflammatory drugs (NSAIDs), interferon, or chemotherapy are options for unresectable or recurrent disease, the appropriate therapeutic approach has not yet been fully elucidated [14].

Carbon ion radiotherapy can precisely irradiate the target lesion, thereby reducing its adverse effects on the adjacent intestinal tracts. Photon beam sensitivity varies with the type of tumor, whereas carbon ion radiotherapy, which uses carbon (C) molecules, can be expected to have potent effects on all types of tumor, irrespective of sensitivity [15]. The finding that carbon ion radiotherapy was effective for recurrent desmoid tumor of the abdominal wall in this patient will lead to an increase in treatment options and thus have important implications for patients suffering from this disease and for the physicians treating them. In addition, this is the first report of a case of desmoid tumor successfully treated with carbon ion radiotherapy. We hope that our report will be of some help in determining the treatment strategy for this disease.

## Conclusions

This report presents a first case that the carbon ion radiotherapy has been effective for the unresected desmoid tumor.

## Abbreviations

APC: adenomatous polyposis coli
cm: centimeter
CT: Computed tomography
FAP: Familial adenomatous polyposis
Gy: Gray
NSAIDs: Nonsteroidal anti-inflammatory drugs

## Footnotes

1 The carbon ion beam dose is reported in Gy (RBE), which is defined as the physical dose multiplied by the RBE of the protons or carbon ions. For example, 2 Gy (RBE) of carbon ion radiotherapy is biologically equivalent to 2 Gy of photon radiotherapy. Biological effects of carbon ion radiotherapy at HIBMC have been evaluated in vitro and in vivo, and the RBE values for carbon ion irradiation were determined to be 2–3.7 (depending on the depth in the spread-out Bragg peaks) [16].
