# Erratum to: Carbon ion radiotherapy for desmoid tumor of the abdominal wall: a case report

**DOI:** 10.1186/s12957-017-1154-z

**Published:** 2017-05-03

**Authors:** Takuya Nagata, Yusuke Demizu, Tomoyuki Okumura, Shinichi Sekine, Naoki Hashimoto, Nobukazu Fuwa, Tomoaki Okimoto, Yutaka Shimada

**Affiliations:** 1 Department of Surgery and Science, Graduate School of Medicine and Pharmaceutical Sciences for Research, University of Toyama, 2630 Sugitani, Toyama-shi, Toyama 930-0194 Japan; 2 Department of Radiology, Hyogo Ion Beam Medical Center, 1-2-1 Koto, Shingu-cho, Tatsuno-shi, Hyogo, Japan; 3 Department of Radiation Oncology, Kobe Minimally Invasive Cancer Center, Kobe University Hospital 7-5-2 Kusunoki-cho, Chuo-ku, Kobe City, Hyogo Prefecture, Japan; 4 Department of Radiation Oncology, Ise Red Cross Hospital, 471-2 Funae 1 choume, Ise-shi, Mie, Japan; 5 Department of Nanobio Drug Discovery, Graduate School of Pharmaceutical Sciences, Kyoto University, Yoshida-honmachi, Sakyo-ku, Kyoto 606-8501, Japan

## Erratum

Upon publication of the original article [1], the authors mistakenly referred to their experimental technique as “Carbon ion radiotherapy”. This was incorrect; the technique should have been referred to as “Proton beam therapy”. All authors of the original manuscript have understood and agreed to this correction additionally the scientific conclusions of this paper remain unchanged.

## References

[1] Nagata T, Demizu Y, Okumura T, Sekine S, Hashimoto N, Fuwa N, Okimoto T, Shimada Y (2016). Carbon ion radiotherapy for desmoid tumor of the abdominal wall: a case report. World J Surg Oncol.

